# Pharmacodynamic characteristics and influencing factors of tapentadol for chronic pain relief under dose titration

**DOI:** 10.3389/fpain.2024.1474529

**Published:** 2025-01-17

**Authors:** Liang Xin, Haoxiang Zhu, Suping Niu, Xie Han, Hongxian Pang, Jiangfan Li, Ye Hu, Xuhong Wang, Lujin Li, Yi Fang

**Affiliations:** ^1^ Medical Institution Conducting Clinical Trials for Human Used Drug, Beijing Luhe Hospital, Capital Medical University, Beijing, China; ^2^ Center for Pharmacometrics, Shanghai University of Traditional Chinese Medicine, Shanghai, China; ^3^ State Key Laboratory of Integration and Innovation of Classic Formula and Modern Chinese Medicine, Shanghai University of Traditional Chinese Medicine, Shanghai, China; ^4^ Clinical Trial Institution, Peking University People’s Hospital, Beijing, China; ^5^ Department of Pharmacy, Nanjing Drum Tower Hospital, Nanjing, Jiangsu, China; ^6^ Clinical Pharmacology Department, Beijing Luhe Hospital, Capital Medical University, Beijing, China

**Keywords:** chronic pain, opioids, tapentadol, model-based meta-analysis, pharmacodynamic

## Abstract

**Objective:**

The aim of this study was to establish a pharmacodynamic model of tapentadol analgesia under dose titration conditions, to quantitatively analyze the time-effect relationship of the drug, and to identify relevant influencing factors. This model is intended to provide a pharmacodynamic reference for designing rational tapentadol dose titration schemes in clinical research.

**Methods:**

Randomized controlled trials assessing the efficacy of tapentadol in the management of chronic pain were retrieved from public databases (PubMed and EMBASE). A time-effect relationship model of the percent change in Numerical Rating Scale (NRS) scores post-tapentadol intervention from baseline was constructed, along with a covariate model to identify factors significantly impacting the analgesic effects of tapentadol. Potential influencing factors that were clinically significant but not included in the final covariate model were examined for their impact trends on tapentadol analgesia through subgroup analysis.

**Results:**

A total of 16 studies involving 4,508 participants were included in the analysis. Covariate analysis indicated that age significantly affected the maximum reduction in NRS scores following tapentadol treatment, with the reduction rate being 40.9% for patients aged 45 and 60.7% for those aged 65, suggesting that older patients have a higher demand for pain relief. Furthermore, studies published after 2014 and placebo-controlled trials showed a slower rate of NRS reduction, indicating a more cautious approach to tapentadol dosing titration post the U.S. opioid crisis and in placebo-controlled contexts. Additionally, subgroup analysis suggested that higher titration doses, higher baseline NRS levels, the use of extended-release tapentadol, and a smaller proportion of male participants were trends associated with better analgesic effects, although the differences were not statistically significant. Moreover, the study found that tapentadol was significantly more effective in treating lower back pain compared to non-lower back pain.

**Conclusion:**

This research successfully developed a pharmacodynamic model for dose-titrated tapentadol administration, which can simulate the temporal changes in analgesic effects of tapentadol across different clinical scenarios. This model can guide the formulation of dosing titration protocols for tapentadol in clinical research.

**Systematic Review Registration:**

https://inplasy.com/inplasy-2024-5-0014/

## Introduction

Chronic pain, characterized by prolonged pain persisting beyond the typical healing period or for over three to six months, impacts approximately 20%–30% of adults worldwide. Symptoms are varied and complex, encompassing persistent or intermittent pain—often described as shooting, burning, aching, or electrical—along with associated fatigue, sleep disturbances, reduced appetite, and mood changes such as depression and anxiety (1). The condition poses a considerable socio-economic challenge, affecting individual quality of life and leading to significant societal costs due to healthcare expenditures and lost productivity. For instance, in the United States, the estimated annual cost of chronic pain ranges from $560 billion to $635 billion (2). Management of chronic pain currently necessitates personalized, multi-modal, and interdisciplinary approaches, including pharmacological treatments, acupuncture, and potentially surgical interventions (3).

Opioids are one of the longstanding keystones in chronic pain management, functioning by binding to opioid receptors in the brain, spinal cord, and elsewhere, consequently diminishing pain perception (4). They exhibit efficacy in managing severe and various types of pain, including cancer-related, postoperative, and chronic non-cancer pain. Despite their effectiveness, opioids carry significant side effects such as constipation, nausea, sedation, and respiratory depression. Prolonged use may result in tolerance, necessitating higher doses for equivalent pain relief, and can lead to physical dependence (5).

Tapentadol, a centrally acting opioid analgesic belonging to the benzenoid class, exhibits a dual mode of action. It functions as both an agonist of the *μ*-opioid receptor and a norepinephrine reuptake inhibitor (NRI) (6). Its dual action enhances its effectiveness in relieving moderate to severe pain in both acute and chronic musculoskeletal conditions, such as those resulting from injuries, surgeries, and long-term degenerative diseases. With a potency that lies between that of tramadol and morphine, tapentadol stands out due to its analgesic efficacy, which is comparable to that of oxycodone (7). Notably, it achieves this level of efficacy while causing fewer side effects, making it a potent and relatively safer choice for pain management (8).

Despite its FDA approval over a decade ago and a robust clinical profile, several practical issues concerning the application of tapentadol have yet to be resolved. Tapentadol is administered as a titrated dose, with daily dosages ranging from 50 to 500 mg, which should be adjusted until the patient's pain becomes tolerable (9). Determining the precise titration to an appropriate dose for a patient within such a broad range is a common concern for clinicians. Moreover, patients of different ages and those with pain in various anatomical locations exhibit divergent tolerances to pain and thus may require different dosages, necessitating further clarification (10, 11). Additionally, tapentadol is available in immediate-release and extended-release formulations (12). These factors can significantly impact the dose-adjustment strategies for tapentadol. Further research and clarification are needed to optimize the use of tapentadol in clinical practice and improve patient outcomes.

This study is designed to develop a clinical pharmacodynamic model for tapentadol, utilizing a comprehensive analysis of existing literature to accurately delineate the drug's therapeutic profile in clinical settings. The resulting model will establish a reference standard to inform the clinical titration process for tapentadol dosages. Furthermore, the investigation will focus on identifying of factors that affect the drug's efficacy, with the goal of providing a well-founded framework to enhance the precision of tapentadol dosing strategies.

## Methods

### Research criteria and eligibility

The methodology employed for this study involved a systematic search of randomized controlled trials (RCTs) related to the management of chronic pain using tapentadol. The search was performed in the publicly accessible databases of PubMed and EMBASE, with the search timeline extending up to September 30, 2023. The specific search strategy employed, including search terms and their combinations, is thoroughly documented in the Supplementary Section of this paper. The process of literature selection, data extraction, and analysis in this study strictly adheres to the protocols delineated in the Preferred Reporting Items for Systematic Reviews and Meta-Analyses (PRISMA) guidelines, ensuring the transparency and reproducibility of our research.

The primary outcome measure in this study was the reduction in the Numerical Rating Scale (NRS) from baseline. The inclusion criteria were as follows: (1) Randomized controlled clinical trials (RCTs); (2) Studies where tapentadol monotherapy was used as the treatment; (3) Studies involving subjects experiencing various types of chronic pain and diagnosed as functional capacity levels I–III; (4) Studies that reported NRS scores at specific time points for chronic pain; (5) Studies involving participants aged 18 years or older.

The exclusion criteria included the following: (1) Studies involving subjects without a washout period before enrollment. Since tapentadol is primarily used as a second-line treatment, participants typically have received other opioid treatments before joining the study. Without a washout period, previous medications could potentially affect the efficacy assessment of tapentadol; (2) Cancer pain research; (3) Studies where chronic pain was not accompanied by reported NRS baseline values; (4) Studies using a randomized withdrawal design; (5) Crossover study designs that did not report data from the first cycle; (6) Studies involving subjects with concurrent psychiatric disorders.

### Data extraction and quality assessment

Data extraction for this study was performed using Microsoft Excel (version 16.38). The extracted data encompassed several categories: (1) Basic literature information, including authors, year of publication, and clinical trial registration number; (2) Trial details such as the formulation and dosage of tapentadol, the etiology of pain, sample size, trial duration, the use of blinding and placebo control; (3) Participant characteristics, which included the mean age, gender ratio, and baseline NRS score; (4) Outcome measures, specifically the change in NRS score from baseline at each visit. To ensure the accuracy and reliability of the data extraction process, it was independently conducted by two researchers. Any discrepancies between their findings were resolved through discussion with a third researcher. Graphical data were extracted using Engauge Digitizer software (version 4.1). If the extraction error between the two researchers exceeded 2%, the data in question were re-extracted, and the average value of the two extracts was used for further analysis.

The risk of bias (RoB) for each included randomized controlled trial (RCT) was independently examined by two researchers utilizing the Cochrane RoB2 tool (13). This tool, specifically the RoB2_IRPG_beta_v9.xlsm, was employed to assess potential bias in five key areas: the randomization process, deviation from intended interventions, missing outcome data, measurement of the outcome, and selection of the reported result. The RoB for each category was designated as “low” if there was a low risk of bias, “high” if there was a high risk of bias, or “unclear” if there was insufficient information or uncertainty about potential bias. In the event of discrepancies in the RoB2 assessments between the two primary researchers, a third researcher was consulted to review the assessments and make the final decision.

### Model establishment and evaluation

A time-effect model was developed using the change in NRS from baseline as the efficacy indicator. Influencing factors such as dosage, formulation, administration frequency, baseline NRS values, male ratio, and year of publication (before or after 2014) were considered, along with whether a placebo control was used. The year 2014 was a critical turning point in the U.S. opioid crisis, characterized by a significant increase in overdose deaths, heightened media attention, proactive interventions from governmental and regulatory bodies, and the beginning of legal challenges against pharmaceutical companies. In response to the crisis, the FDA implemented numerous warnings and updated guidelines that emphasized the need for clearer medication labeling. These concerted efforts increased public awareness of the risks associated with opioids and enhanced strategies to address the crisis. This, in turn, influenced the methodologies used in subsequent clinical research (14–16).

This model quantified the relationship between the change in NRS from baseline and time, and examined the influencing factors. Detailed methods are available in the Supplementary Materials.

## Results

### Characteristics of the selected studies

**Figure 1:**
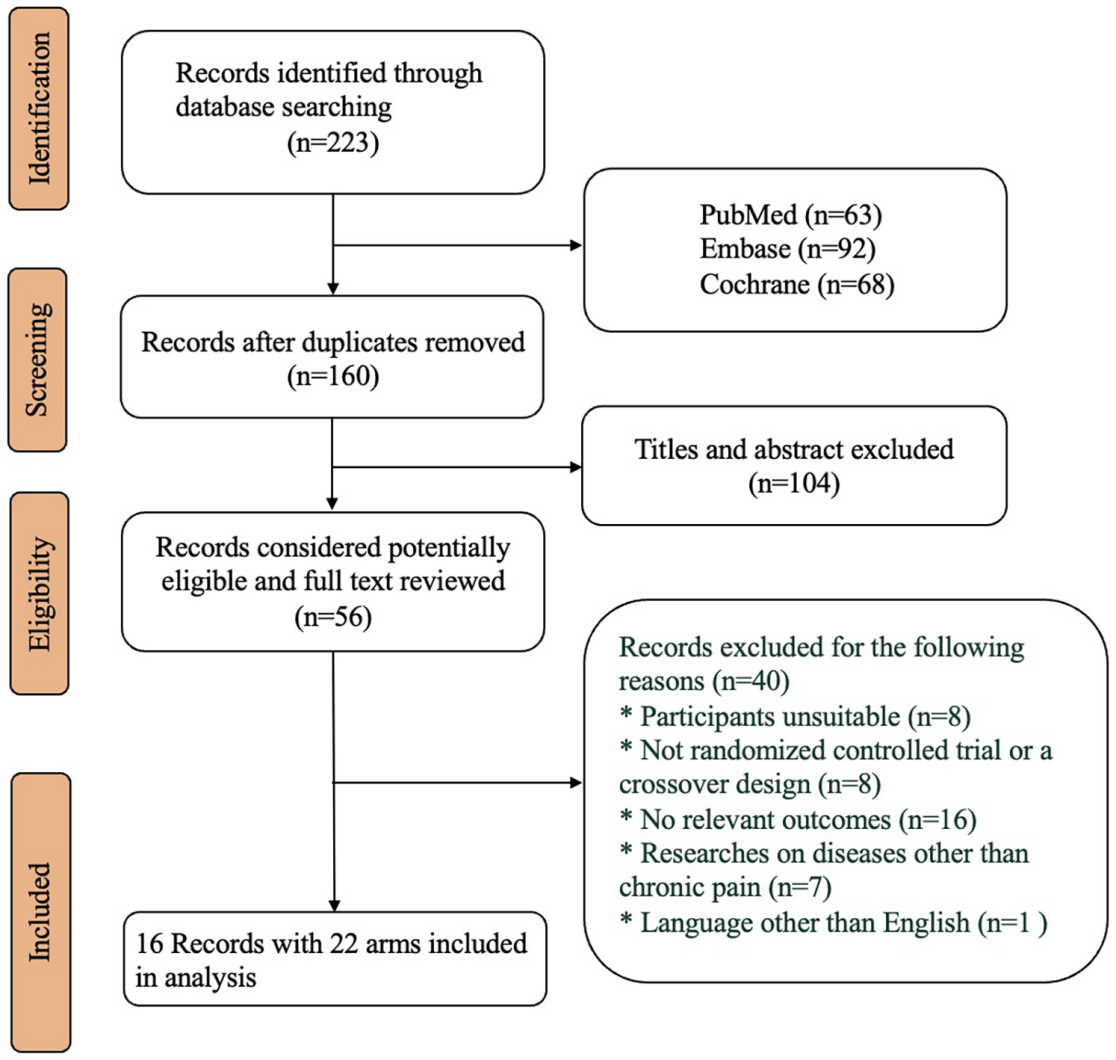
Flowchart of literature screening.

A total of 223 articles were retrieved, of which 16 articles (representing 7.2% of the total) were included in the final analysis. These articles comprised 22 treatment arms involving 4,508 participants treated with tapentadol. All the trials were designed using dose titration. The mean age of the participants ranged from 41.4 to 65.5 years (with a median of 58.4 years), and the male proportion varied from 36% to 59% (with a median of 42.4%). The average BMI of the participants ranged from 25.2 to 33.6 kg/m^2^ (with a median of 31.1 kg/m^2^), and the baseline NRS scores of the participants ranged from 3.9 to 8.4 (with a median score of 7.5). The specific literature screening process can be found in Figure 1, and the included studies and their demographic characteristics are provided in the Supplementary.

The results of the literature quality assessment indicated that four studies were deemed to present a “high” risk, two studies were judged to have a “medium” risk, and 14 studies were considered to bear a “low” risk. Detailed assessments of the literature quality can be found in the Supplementary.

### Modelling results

The estimated values of the final model parameters are shown in Table 1. Covariate analysis revealed that age significantly influenced the parameter *E*_max_, while the year of publication (before or after 2014) and the use of a placebo control significantly affected the parameter ET_50_. The expression for the final covariate model can be seen in Equations 1, 2. Specifically, when the age of the participant was 59 years, the maximum reduction in the NRS from baseline following tapentadol intervention was 56.7%. For participants aged 45 and 65 years, the maximum reductions in the NRS from baseline post-tapentadol intervention were 40.6% and 63.7%, respectively. The latter represented a 23.1% higher reduction compared to the former. When the year of publication was before 2014 (i.e., PY = 0 in Equation 2) and the trial was not placebo-controlled (i.e., Placebo = 0 in Equation 2), the ET_50_ value was 0.984 weeks. When the year of publication was after 2014 (i.e., PY = 1 in Equation 1), the ET_50_ value for tapentadol extended by 1.71 weeks. In placebo-controlled trials (i.e., Placebo = 1 in Equation 2), the ET_50_ value for tapentadol extended by 1.19 weeks.

(1) $$E_{\max}=-56.7\times {\left(\frac{\mathrm{Age}}{59}\right)}^{1.21}$$

(2) $$ET_{50}=0.984\times (1+1.74\times PY)+1.19\times \mathrm{PLACEBO}$$

**Table 1:** Model prediction parameters and bootstrap results.

	Estimate (RSE%)	Bootstrap (924/1,000)
Fixed effect		
*θ* (*E*_max_)	−56.7 (5.9)	−56.41 (−62.45, −51.55)
*θ* (ET_50_, week)	0.984 (13.9)	0.99 (0.63, 1.34)
*θ* (ublished after 2014 on ET_50_)	1.74 (35.8)	1.73 (0.33, 3.15)
*θ* (Placebo on ET_50_)	1.19 (21.0)	1.17 (0.47, 1.92)
*θ* (Age on *E*_max_)	1.21 (31.2)	1.27 (0.23, 2.20)
Inter-study variability		
*θ* (*E*_max_, %)	13.8 (15.2)	12.3 (8.5, 17.6)
*η* (ET_50_, %)	28.7 (30.2)	24.8 (0, 42.0)
Residual error		
*ε*,%	118.7 (11.3)	119.3 (88.4, 142.9)

A 1,000 bootstrap repeated sampling was used for internal verification, and 924 parameter estimations were successful. By analyzing the 924 results, the median parameter estimation obtained by bootstrap was consistent with that of the original dataset.

**Figure 2:**
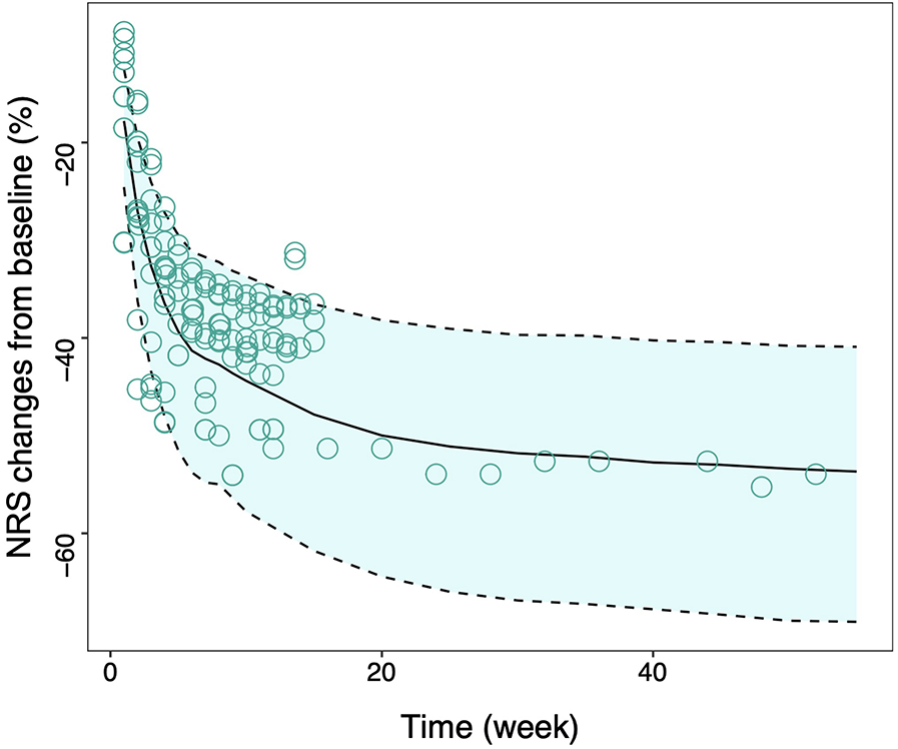
Visual inspection of final model. Model predicted 95% CI of tapentadol response, the solid line is the median value of predicated data and the dotted lines are the model predicted 2.5th and 97.5th percentiles of efficacy. The points represent the observed data and the symbol size is proportional to the sample size. NRS, Numerical Rating Scale; CI, confidence interval.

The final model exhibited a robust goodness-of-fit without an evident bias, as shown in the supplementary. An internal validation was conducted utilizing 1,000 iterations of bootstrap resampling, which resulted in successful parameter estimation in 924 iterations. Upon analyzing the results from 924 iterations, it was found that the median of the parameter estimates derived from the bootstrap was in alignment with the parameter estimates from the original dataset (Table 1). This correspondence attests to the stability of the model parameter estimation. The VPC results (Figure 2) revealed that the 95% confidence interval (CI) of the model predictions encompassed the majority of the observed values, suggesting the model's strong predictive performance.

### Typical efficacy of tapentadol in trial participants of different ages

**Figure 3:**
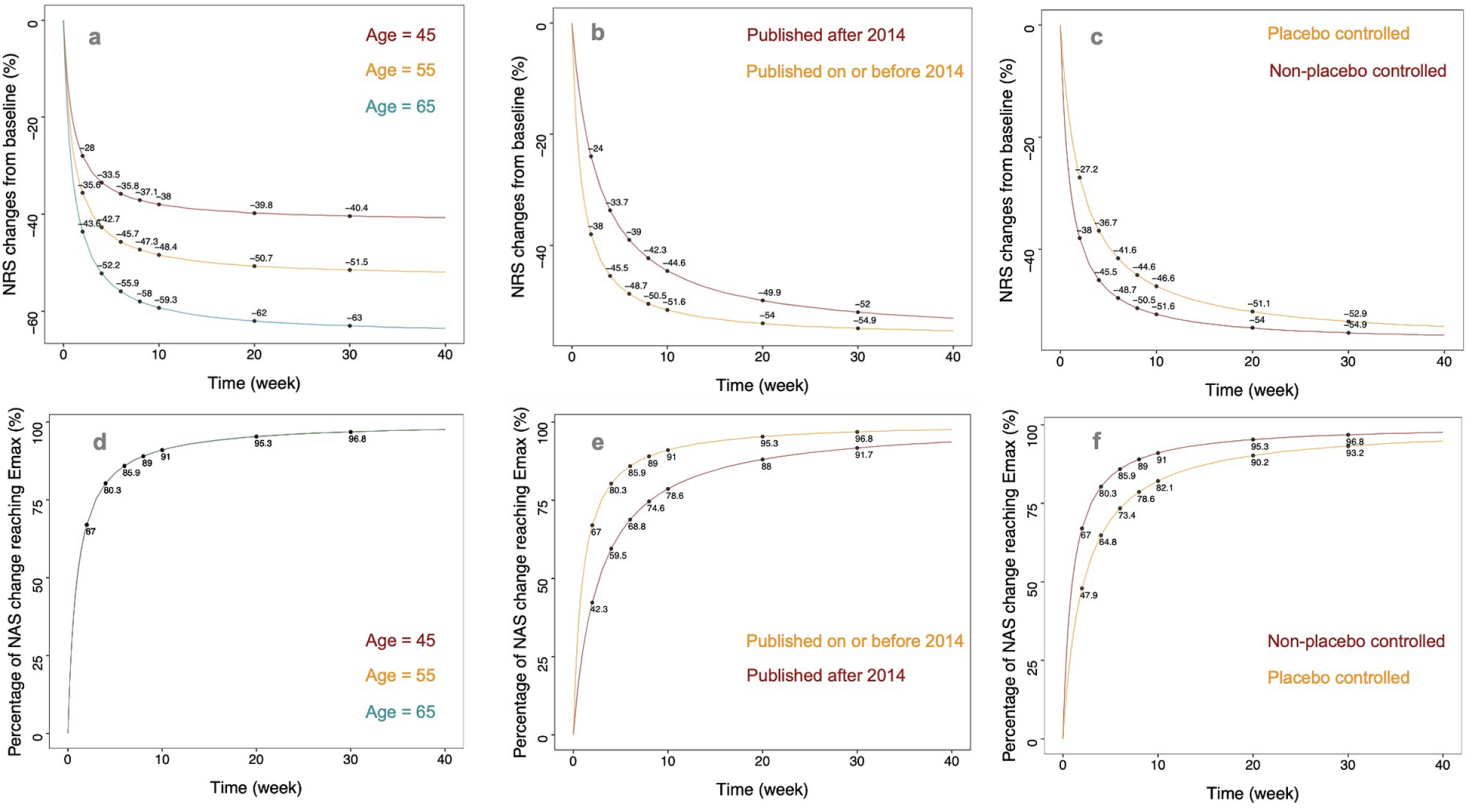
The typical predicted time course of tapentadol response. **(a)** Illustration of predicted typical values of NRS relative percentage change from baseline in non-placebo controlled trials published before 2014 for subjects at different ages (45, 55, 65 years). **(b)** Typical value predictions of NRS relative percentage change from baseline in non-placebo controlled trials with an average age of 59 years (median age in the literature) for publications before and after 2014. **(c)** Typical value predictions of NRS relative percentage change from baseline in trials with an average age of 59 years and published before 2014, comparing placebo-controlled and non-placebo controlled experiments. **(d)** Illustration of predicted typical values of NRS change from baseline to Emax percentage in non-placebo controlled trials published before 2014 for subjects at different ages **(e)** Typical value predictions of NRS change from baseline to Emax percentage in non-placebo controlled trials with an average age of 59 years, differentiating publications before and after 2014. **(f)** Typical value predictions of NRS change from baseline to Emax percentage in trials with an average age of 59 years and published before 2014, comparing placebo-controlled and non-placebo controlled experiments.

Based on the final model, we simulated the change rate in NRS from baseline following tapentadol intervention for trial participants at three age levels (45, 55, and 65 years). During the simulation, the year of publication was fixed as post-2014, and the trial design was set to placebo-controlled. Taking the age of 55 years as an example, the change rates in NRS from baseline at 1 week, 4 weeks, 8 weeks, 12 weeks, and 16 weeks post-treatment were 10.6%, 26.3%, 35.0%, 39.3%, and 41.8% respectively. These represented 17.1%, 50.5%, 67.1%, 75.4%, and 80.2% of their *E*_max_ value (52.1%). When the ages of the trial participants were 45, 55, and 65 years, the change rates in NRS from baseline at the 16th week were 32.8%, 41.8%, and 51.2% respectively (Figures 3a,d).

### Typical efficacy of tapentadol for different years of publication

We simulated the change rate in NRS from baseline following tapentadol intervention for studies published before and after 2014. During the simulation, the age was fixed at 55 years, and the trial design was set to placebo-controlled. For studies published before 2014, the change rates in NRS from baseline at 1 week, 4 weeks, 8 weeks, 12 weeks, and 16 weeks post-treatment were 16.4%, 33.7%, 41.0%, 44.1%, and 45.9%, respectively. These represented 31.4%, 64.7%, 78.7%, 84.6%, and 88.1% of their *E*_max_ value (52.1%). The efficacy plateau for participants in studies published before 2014 appeared approximately at the 8th week. For studies published after 2014, the change rates in NRS from baseline at 1 week, 4 weeks, 8 weeks, 12 weeks, and 16 weeks post-treatment were 10.6%, 26.3%, 35.0%, 39.3%, and 41.8%, respectively. These represented 17.1%, 50.5%, 67.1%, 75.4%, and 80.2% of their *E*_max_ value (52.1%). The efficacy plateau for participants in studies published after 2014 appeared approximately at the 16th week (Figures 3b,e).

### Typical efficacy of tapentadol under different trial designs

This study found that the design of placebo control significantly impacts the efficacy of tapentadol. We simulated the typical efficacy values of tapentadol under both placebo-controlled and non-placebo-controlled conditions. During the simulation, the age was fixed at 55 years, and the year of publication was set as post-2014. In non-placebo-controlled studies, the change rates in NRS from baseline at 1 week, 4 weeks, 8 weeks, 12 weeks, and 16 weeks post-treatment were 14.0%, 31.0%, 38.9%, 42.4%, and 44.5%, respectively. These represented 26.9%, 59.5%, 74.7%, 81.4%, and 85.4% of their *E*_max_ value (52.1%). The efficacy plateau for participants in non-placebo-controlled studies appeared approximately at the 12th week. In placebo-controlled studies, the change rates in NRS from baseline at 1 week, 4 weeks, 8 weeks, 12 weeks, and 16 weeks post-treatment were 10.6%, 26.3%, 35.0%, 39.3%, and 41.8%, respectively. These represented 17.1%, 50.5%, 67.1%, 75.4%, and 80.2% of their *E*_max_ value (52.1%). The efficacy plateau for participants in placebo-controlled studies appeared approximately at the 16th week (Figures 3c,f).

### Other potential influencing factors on the efficacy of tapentadol

**Figure 4:**
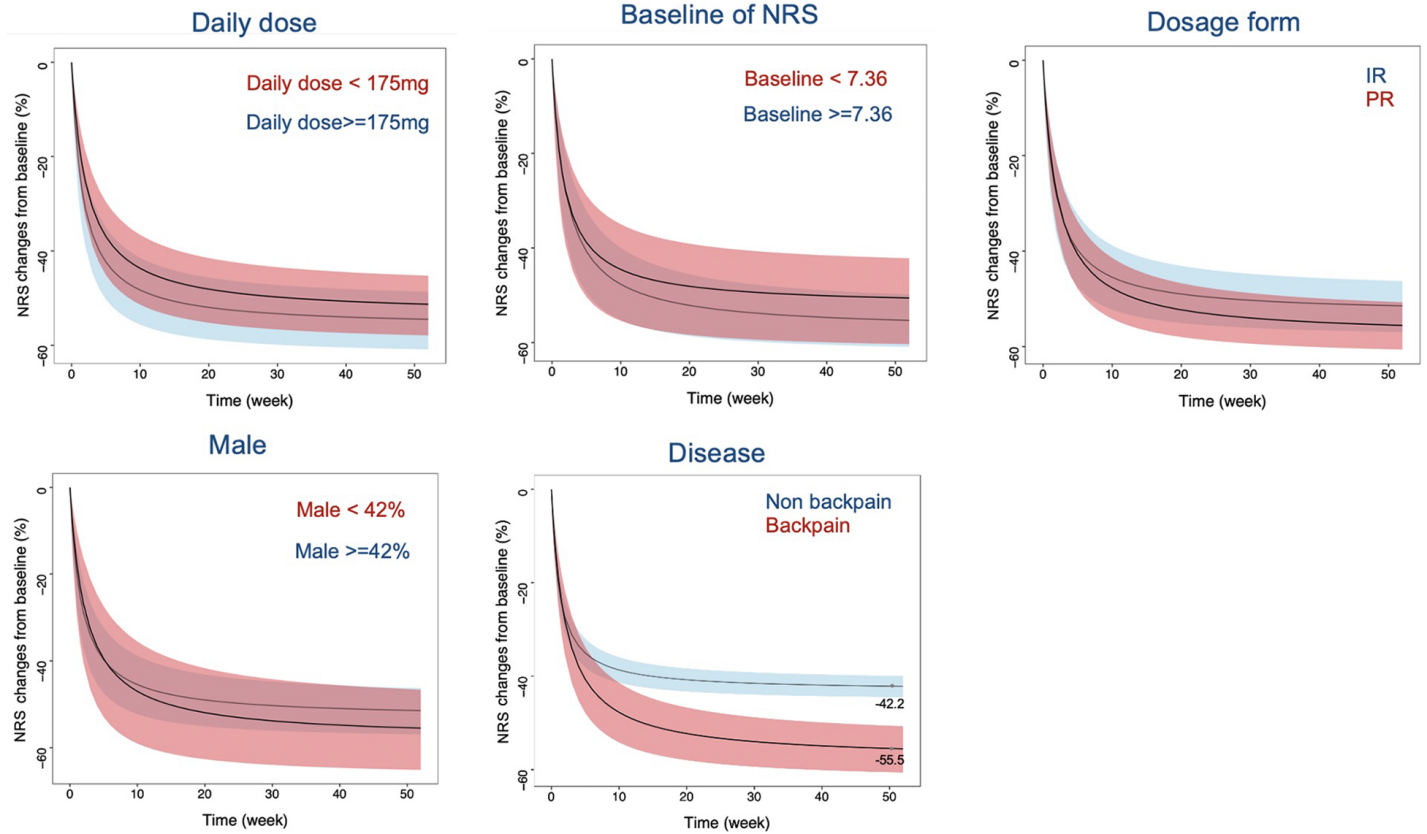
Typical values and 95% CI simulation of NRS relative percentage change from baseline across different daily doses, NRS baseline, formulations, gender ratios, and disease types. The black curve represents the typical response of tapentadol, while the shaded area represents the 95% CI. The colors differentiate different influencing factors. NRS, Numerical Rating Scale; CI, confidence interval; IR, Immediate release; PR, prolonged release.

This study investigated the trend of other factors influencing the efficacy of tapentadol, aside from covariates, through subgroup analysis. In the subgroup analysis, the age of participants was adjusted to 55 years, the year of publication was adjusted to post-2014, and the trial design was adjusted to placebo-controlled to eliminate the influence of heterogeneity at the covariate level on the results of the subgroup analysis. The results showed that a higher titrated doses of tapentadol, a higher baseline NRS level of the subjects, a sustained-release formulation of tapentadol, and a smaller proportion of male subjects tended to have better efficacy. However, due to large variations, the differences were not statistically significant. For example, at 24 weeks with other factors controlled, the percentage change in NRS from baseline varied significantly across different conditions. Tapentadol doses below 175 mg resulted in a change of −48.9 (95% CI: −55.9 to −42.5), while doses of 175 mg or greater showed a change of −52.6 (95% CI: −59.3 to −46.4). Similarly, baseline NRS values below 7.36 led to a change of −48.7 (95% CI: −58.9 to −39.9), compared to −52.9 (95% CI: −59.2 to −46.6) for values of 7.36 or higher. Immediate-release (IR) formulations had a change of −48.7 (95% CI: −54.2 to −43.5), whereas extended-release (ER) formulations showed −54.6 (95% CI: −64.1 to −45.0). When the male ratio was below 42%, the change was −52.8 (95% CI: −63.3 to −42.9), vs. −49.6 (95% CI: −55.6 to −43.9) for a ratio above 42%. Additionally, non-back pain resulted in a change of −41.2 (95% CI: −43.6 to −38.8), compared to −53.1 (95% CI: −58.7 to −47.7) for back pain (Figure 4). These variations underline the influence of dosage, baseline severity, formulation, gender distribution, and type of pain on the effectiveness of tapentadol. Moreover, we found significant differences in the efficacy of tapentadol for different pain locations. The efficacy of tapentadol for lower back pain was significantly better than for non-back pain. For instance, at 52 weeks, the NRS reduction rates for the two groups differed by about 15%.

## Discussion

The pharmacodynamic model developed in this investigation offers a precise representation of tapentadol's therapeutic efficacy within a clinical context, and serves as a guide for the titration of its dosage. Efficacy was assessed by measuring the reduction in NRS scores from baseline, a reliable endpoint for quantifying tapentadol's analgesic impact. The data reveal that the maximal observed decrease in NRS scores post-tapentadol administration was 56.7%, indicating the peak demand for pain relief as gauged by this metric. Our analysis also demonstrated a clear age-related decline in pain tolerance; specifically, patients at the ages of 45 and 65 experienced maximum NRS reductions of 40.9% and 60.7%, respectively. This trend may be attributed to the age-related decline in endogenous analgesic mechanisms and the diminished pain modulation capacity in older adults, factors that have been associated with the increased incidence and intensity of chronic pain with age (17). Moreover, the influence of age on the pharmacokinetics and pharmacodynamics of analgesic drugs has been well-documented, highlighting the critical need to factor in patient age when prescribing tapentadol (18).

This study found that the onset of action for tapentadol occurred approximately within one week, with patients achieving 50% of the maximum reduction rate in Numerical Rating Scale (NRS) scores at this time point. Moreover, the study identified that the year of publication and whether the study was placebo-controlled significantly influenced the onset of action of tapentadol. Specifically, research published prior to 2014 reported an average onset time of approximately 0.984 weeks, compared to 1.712 weeks for studies released after 2014. When examining post-2014 studies, those without a placebo control initiated action at 1.712 weeks, whereas placebo-controlled trials reported a longer onset time of 2.902 weeks. This variation can be attributed to tapentadol's dosing regimen, which involves titration; hence, the onset time is inherently linked to both the dosage amount and the titration pace. After 2014, clinical trials involving tapentadol saw a deliberate reduction in dosing and a more gradual approach to titration. Placebo-controlled studies necessitate careful titration to avoid confounding effects that could mask the true efficacy of the drug. Thus, they exhibit slower dosing and titration schedules compared to trials without a placebo group (19).

In clinical practice, achieving a reduction of at least 30% in NRS score is widely accepted as indicative of clinically meaningful pain relief (20). This study utilizes this benchmark to model the duration needed to attain such a reduction in NRS scores across various scenarios (refer to Supplementary Materials), providing guidance for clinicians when tailoring dosage regimens. Analysis of non-placebo-controlled studies published post-2014 reveals that participants aged 45 required approximately 7 weeks to achieve a 30% NRS score reduction, whereas those aged 65 required only 2.5 weeks. Preclinical studies have also found that elderly mice are more sensitive to the efficacy of opioids (21). Additionally, for studies with a mean participant age of 55, conducted after 2014, it took 5.3 weeks to achieve this level of reduction in placebo-controlled studies, compared to 3.7 weeks in non-placebo-controlled studies. For non-placebo-controlled trials with participants averaging 55 years of age, the duration to reach a 30% reduction in NRS scores was shorter at 1.4 weeks in studies published before 2014, compared to 3.7 weeks in those published afterwards.

Our subgroup analysis aimed to assess the influence of clinically relevant variables not accounted for as covariates on the effectiveness of tapentadol. We noted a pattern where subjects with higher baseline NRS scores, those on higher titrated doses, and groups with a smaller proportion of male participants receiving extended-release tapentadol tended to experience improved pain relief. However, this trend did not reach statistical significance. The expectation that extended-release formulations, which maintain steadier plasma drug concentrations, would provide enhanced analgesia is supported by findings from multiple studies (22, 23). Additionally, prior research suggests that women, in comparison to men, may have lower pain thresholds and a lower tolerance to pain, potentially due to hormonal differences (24). Our research also indicates that the efficacy of analgesics is dependent on the pain's location, with back pain responding more favorably than other types of pain. Since back pain generally stems from musculoskeletal problems like muscle strains, ligament sprains, or disc degeneration, these conditions might be more sensitive to analgesic treatment. However, there is a dearth of literature comparing the analgesic effects on different pain sites. Given the small number of back pain trials included in our study, further research is warranted to corroborate these findings. However, traditional meta-analyses, like the study conducted by Santos et al. (25), cannot analyze the full time course of drug effects or assess the impact of various confounding factors. This limitation results in significant heterogeneity among trials that remains unexplained.

The present study is subject to several limitations. Our efficacy evaluation was solely based on NRS scores. While some studies did report outcomes using the Visual Analogue Scale (VAS), the heterogeneity between VAS and NRS assessments prevented us from integrating these two metrics into a combined analysis. Moreover, existing literature indicates potential racial variations in the analgesic response to opioids for back pain (26), but due to data constraints in the available reports, an examination of racial influences on tapentadol's effectiveness was beyond the scope of this study. Our analysis also relied on synthesized data from the literature, which meant individual patient data was inaccessible, thereby inhibiting the development of a dose-response model for elucidating tapentadol's pharmacodynamic profile. The studies we included were all conducted in the United States, exhibiting high homogeneity. However, they cannot reflect the differences in efficacy caused by variations in different regions. Currently, the mainstream clinical treatment for pain is multimodal therapy, which includes not only medication but also acupuncture and moxibustion, rehabilitation exercise, and other treatment options. This study only provides a reference for the efficacy of medication treatment. When the model is used for clinical reference, it should be noted that combining it with other therapies may lead to inconsistent treatment outcomes. Finally, the exclusion of non-English language studies could have introduced publication bias into our findings.

## Conclusion

Utilizing a comprehensive array of literature, this study established a time-effect model to characterize the analgesic properties of tapentadol. This model delineates the drug's efficacy profile in diverse clinical contexts. The insights gleaned from this model offer a valuable pharmacodynamic framework that can inform the development of dosing titration strategies for tapentadol in clinical settings and forthcoming research trials.

## Data Availability

The original contributions presented in the study are included in the article/Supplementary Material, further inquiries can be directed to the corresponding authors.
