# Supplementary material for: Pharmacodynamic characteristics and influencing factors of tapentadol for chronic pain relief under dose titration
Source: Front Pain Res (Lausanne). 2025 Jan 17;5:1474529. doi: 10.3389/fpain.2024.1474529 (PMC11782195; doi:10.3389/fpain.2024.1474529)
Supplement: Supplementary file 1 [file Datasheet1.docx]

Catalog

[PubMed search query 2](#_Toc185390086)

[Cochrane search query 3](#_Toc185390087)

[Embase search query 3](#_Toc185390088)

[Model establishment and evaluation 4](#_Toc185390089)

[Table S1: Baseline characteristics and basic information of the included literature 7](#_Toc185390090)

[Reference included 8](#_Toc185390091)

[Figure S1. Risk of Bias 10](#_Toc185390092)

[Figure S2. Goodness of fit 10](#_Toc185390093)

[Table S2. Typical time (week) to achieve -30% efficacy 11](#_Toc185390094)

[Reference 11](#_Toc185390095)

| Search number | Query | Results |
| --- | --- | --- |
| 11 | ((((tapentadol[MeSH Terms]) OR (nucynta[MeSH Terms])) AND (((ache[MeSH Terms]) OR (suffering[MeSH Terms])) OR (pain[MeSH Terms]))) AND (clinical trial[Filter])) AND (("1900/01/01"[Date - Publication] : "2023/09/30"[Date - Publication])) | 63 |
| 10 | ("1900/01/01"[Date - Publication] : "2023/09/30"[Date - Publication]) | 36,183,484 |
| 9 | (((tapentadol[MeSH Terms]) OR (nucynta[MeSH Terms])) AND (((ache[MeSH Terms]) OR (suffering[MeSH Terms])) OR (pain[MeSH Terms]))) AND (clinical trial[Filter]) | 63 |
| 8 | ((tapentadol[MeSH Terms]) OR (nucynta[MeSH Terms])) AND (((ache[MeSH Terms]) OR (suffering[MeSH Terms])) OR (pain[MeSH Terms])) | 273 |
| 7 | (tapentadol[MeSH Terms]) OR (nucynta[MeSH Terms]) | 415 |
| 6 | ((ache[MeSH Terms]) OR (suffering[MeSH Terms])) OR (pain[MeSH Terms]) | 617,446 |
| 5 | ache[MeSH Terms] | 468,859 |
| 4 | suffering[MeSH Terms] | 617,446 |
| 3 | pain[MeSH Terms] | 468,859 |
| 2 | nucynta[MeSH Terms] | 415 |
| 1 | tapentadol[MeSH Terms] | 415 |

## **PubMed search query**

| Search number | Query | Results |
| --- | --- | --- |
| 9 | #7 and #8 with Cochrane Library publication date Between Jan 1900 and Sep 2023 | 68 |
| 8 | #4 or #5 or #6 | 96234 |
| 7 | #1 or #2 or #3 | 126 |
| 6 | (ache):ti,ab,kw | 740 |
| 5 | (suffering):ti,ab,kw | 21744 |
| 4 | MeSH descriptor: [Pain] explode all trees 75424 | 75424 |
| 3 | (nucynta):ti,ab,kw | 4 |
| 2 | (tapentadol hydrochloride):ti,ab,kw | 42 |
| 1 | MeSH descriptor: [Tapentadol] explode all trees | 85 |

## **Cochrane search query**

## **Embase search query**

| Search number | Query | Results |
| --- | --- | --- |
| 4 | #3 AND 'clinical trial'/de AND [01-01-1900]/sd NOT [01-10-2023]/sd AND [<1966-2023]/py | 100 |
| 3 | #1 AND #2 | 1708 |
| 2 | 'pain'/exp OR 'suffering'/exp OR 'ache' | 1,808,269 |
| 1 | 'tapentadol'/exp OR nucynta:tn | 2399 |

## **Model establishment and evaluation**

Initial exploratory data analysis suggested a pattern where the rate of change in NRS scores from baseline increased as treatment duration extended, eventually plateauing to reflect the maximum efficacy of the drug. This pattern aligned with the characteristic curve of the Emax model ^[1, 2]^ (Equation 1).

E=$\frac{E_{max}\times Time}{{ET}_{50}\times Time}$ Equation 1

In Equation (1), there are two critical parameters. The first, E_max_, represents the maximum effect of the drug, indicating the highest potential level of pain relief. The second, ET_50_, is the time required to achieve half of the maximum effect. This parameter reflects the onset speed of the drug, providing insight into how quickly the drug begins to alleviate pain.

The variability in drug effects across different studies can be characterized as inter-study variability. In this research, this variability is incorporated into the model parameters in the form of an exponential model, as represented in Equation 2. Any unexplained variability is described as residual error and is accounted for using an additive model, as depicted in Equation 3.

**$P_{i}=P_{typical}\times e^{\eta_{i}}$** Equation 2

**$\text{E}_{\text{obs},i,j}=\text{E}_{\text{pre},i,j}+\frac{\varepsilon_{i,j}}{\sqrt{N_{i,j}/100}}$** Equation 3

In Equation (2), P_typical_ represents the typical value of the pharmacodynamic parameter; η_i_ denotes the inter-study variability of the pharmacodynamic parameter, which is assumed to follow a normal distribution with a mean of 0 and a variance of ω^2^. In Equation (3), E_obs,i,j_ signifies the observed drug effect value at the j^th^ observation point in the i^th^ study; E_pre,i,j_ indicates the predicted drug effect value at the j^th^ observation point in the i^th^ study. ε_i,j_ represents the residual error at the j^th^ observation point in the i^th^ study. N_i,j_ is the sample size (normalized to 100) at the j^th^ observation point in the i^th^ study, with ε_i,j_ being adjusted by the inverse square root of the sample size, signifying that larger sample sizes result in smaller residuals. ε_i,j_ is assumed to follow a normal distribution centered at 0, with a variance of σ^2^.

Once the base model has been established, potential influencing factors on model parameters (E_max_ and ET_50_) can be examined. These factors include dosage, formulation, administration frequency, baseline NRS values, male ratio, year of publication, and whether a placebo control was used. Covariates with missing rates below 20% were imputed using the median, while those with rates equal to or exceeding 20% were imputed using multiple imputation.^[2]^ Continuous covariates were introduced through Equations 4 and 5, whereas binary covariates were incorporated through Equation 6 and 7.

$P_{i}=P_{typical}+(COV-{COV}_{median})\times\theta_{cov}$ Equation 4

$P_{i}=P_{typical}\times{(\frac{COV}{{COV}_{median}})}^{\theta_{cov}}$ Equation 5

$P_{i}=P_{typical}+COV\times\theta_{cov}$ Equation 6

$P_{i}=P_{typical}\times(1+COV\times\theta_{cov})$ Equation 7

In Equations 4 to 7, P_i_ symbolizes the model parameters at varying levels of covariates, while P_typical_ denotes the typical values of these model parameters. The term COV stands for the value of the covariate, COV_median_ corresponds to the median value of the continuous covariate, and θ_cov_ signifies the correction coefficient of the introduced covariate on the model parameters.

Each covariate's influence on the model parameters is individually examined. If the reduction in the model's objective function value (OFV) exceeds 3.84 (the boundary value for a chi-square distribution with 1 degree of freedom, P<0.05), the covariate is deemed to have a significant impact on the parameters. All covariates found to have significant effects through this individual screening are then re-evaluated using forward selection and backward elimination methods to confirm the final covariates to be included in the model. The boundary value for the OFV in the forward selection process is set at 3.84 (P<0.05), while the boundary value for the backward elimination process is set at 6.63 (P<0.01).

The final model underwent evaluation through several metrics, including the standard error of model parameters, OFV, goodness-of-fit plots (GOF) ^[3]^, Bootstrap analysis ^[4-5]^, visual predictive check (VPC) ^[6-7]^, and clinical plausibility assessment. Bootstrap analysis served to evaluate model stability by comparing the distribution of model parameters derived from 1000 resampled datasets with those of the original model. The VPC method involved the simulation of 1000 datasets to determine the 97.5^th^ percentile, 50^th^ percentile, and 2.5^th^ percentile of the drug efficacy distribution. These values were then juxtaposed with the actual observed efficacy values to appraise the predictive performance of the model.

The modeling and simulation procedures were executed using NONMEM (Version 7.4; Icon Inc, PA, USA), employing the first-order conditional estimation (FOCE) method for parameter estimation. All statistical analyses and visual representation of the results were carried out with R software (version 4.0.2).

## Table S1: Baseline characteristics and basic information of the included literature

| **Study** | **Age (year)** | **BMI (kg/m^2^)** | **Male (%)** | **NRS score** | **Daily dose (mg)** | **Disease** |
| --- | --- | --- | --- | --- | --- | --- |
| 2010 Marc Afilalo | 58.4 | 33.61 | 37.2 | 7.4 | 299.3 | Osteoarthritis knee pain |
| 2016 Ralf Baron | 58.1 | 29.8 | 40.8 | 3.9 | 378.8 | Low back pain with a neuropathic component |
| 2015 Ralf Baron (arm 1) | 59.2 | 30.4 | 39 | 7.9 | 300 | Low back pain with a neuropathic component |
| 2015 Ralf Baron (arm 2) | 58.5 | 29 | 38.3 | 8.4 | 500 | Low back pain with a neuropathic component |
| 2016 Ralf Baron | 58.1 | 29.8 | 40.8 | 7.7 | 250 | Low back pain with a neuropathic component |
| 2015 Ralf Baron | 58.5 | 29 | 38.3 | 8.4 | 500 | Low back pain with a neuropathic component |
| 2010 Robert Buyk | 49.4 | 32.09 | 39 | 7.5 | 100-250 | Low back pain |
| 2010 Mila S Etropolski (arm 1) | 53.2 | NA | 51.6 | 7.1 | 50-100 | Low back pain |
| 2010 Mila S Etropolski (arm 2) | 54.7 | NA | 44.8 | 7.5 | 100-250 | Low back pain |
| 2009 Martin Hale | 55.9 | 31.8 | 45.4 | 7 | 50 or 100 | Low back pain or OA pain of the knee or hip |
| 2016 Keiichiro Imaka | 65.5 | NA | 53.6 | 5.4 | 20-200 | Chronic malignt tumor-related pain |
|  |  |  |  |  |  |  |
| 2014 M Niesters | 63 | NA | 58.3 | 7.8 | 100-250 | Diabetic polyneuropathy |
| 2017 Alain Serrie | 62.4 | NA | NA | 7.3 | 221.4 | Chronic osteoarthritis knee pain |
| 2012 lo Steigerwald (arm 1) | 64.6 | 31.1 | 36.7 | 7.1 | 50-250 | Chronic low back pain with or without a neuropathic pain Componen |
| 2012 lo Steigerwald (arm 2) | 60.1 | 29.6 | 39 | 7.6 | 50-250 | Chronic low back pain with or without a neuropathic pain componen |
| 2012 lo Steigerwald (arm 3) | 56.6 | 29.3 | 36 | 7.6 | 50-250 | Chronic low back pain with or without a neuropathic pain componen |
| 2016 Y. Tomiga (arm 1) | NA | NA | NA | 7 | 25–250 | Chronic osteoarthritis pain or low back pain |
| 2016 Y. Tomiga (arm 2) | NA | NA | NA | 7 | 25–250 | Diabetic peripheral neuropathy or peripheral herpetic neuralgia |
| 2014 Aaron I. Vinik | 59 | NA | NA | 7.5 | 100-250 | Chronic, painful diabetic peripheral neuropathy |
| 2011 Gary Vorsanger (arm 1) | 51.1 | 32.2 | 45.1 | 7.1 | 284 | Lower back pain or osteoarthritis pain |
| 2011 Gary Vorsanger (arm 2) | 41.4 | 32.3 | 44 | 7.1 | 284 | Lower back pain or osteoarthritis pain |
| 2010 James E. Wild | 56.8 | 31.7 | 42.4 | 7.6 | 78.56 | Low back pain or osteoarthritis pain |

NA: Not Available

## **Reference included**

1. Afilalo M et al. Efficacy and safety of Tapentadol extended release compared with oxycodone controlled release for the management of moderate to severe chronic pain related to osteoarthritis of the knee: a randomized, double-blind, placebo- and active-controlled phase III study. Clin Drug Investig. 2010;30(8):489-505. doi: 10.2165/11533440-000000000-00000. PMID: 20586515.
2. Baron R et al. Tolerability, Safety, and Quality of Life with Tapentadol Prolonged Release (PR) Compared with Oxycodone/Naloxone PR in Patients with Severe Chronic Low Back Pain with a Neuropathic Component: A Randomized, Controlled, Open-label, Phase 3b/4 Trial. Pain Pract. 2016 Jun;16(5):600-19. doi: 10.1111/papr.12361. Epub 2015 Nov 11. PMID: 26554630.
3. Baron R et al. Effectiveness and Tolerability of a Moderate Dose of Tapentadol Prolonged Release for Managing Severe, Chronic Low Back Pain with a Neuropathic Component: An Open-label Continuation Arm of a Randomized Phase 3b Study. Pain Pract. 2015 Jun;15(5):471-86. doi: 10.1111/papr.12199. Epub 2014 Apr 18. PMID: 24750558.
4. Baron R et al. Effectiveness of Tapentadol Prolonged Release (PR) Compared with Oxycodone/Naloxone PR for the Management of Severe Chronic Low Back Pain with a Neuropathic Component: A Randomized, Controlled, Open-Label, Phase 3b/4 Study. Pain Pract. 2016 Jun;16(5):580-99. doi: 10.1111/papr.12308. Epub 2015 Jun 12. PMID: 26095455.
5. Baron R et al. Effectiveness of Tapentadol Prolonged Release (PR) Compared with Oxycodone/Naloxone PR for the Management of Severe Chronic Low Back Pain with a Neuropathic Component: A Randomized, Controlled, Open-Label, Phase 3b/4 Study. Pain Pract. 2016 Jun;16(5):580-99. doi: 10.1111/papr.12308. Epub 2015 Jun 12. PMID: 2609545
6. Buynak R et al. Efficacy and safety of tapentadol extended release for the management of chronic low back pain: results of a prospective, randomized, double-blind, placebo- and active-controlled Phase III study. Expert Opin Pharmacother. 2010 Aug;11(11):1787-804. doi: 10.1517/14656566.2010.497720. Erratum in: Expert Opin Pharmacother. 2010 Nov;11(16):2773. PMID: 20578811.
7. Etropolski MS, Okamoto A, Shapiro DY, Rauschkolb C. Dose conversion between tapentadol immediate and extended release for low back pain. Pain Physician. 2010 Jan-Feb;13(1):61-70. PMID: 20119464.
8. Hale M, Upmalis D, Okamoto A, Lange C, Rauschkolb C. Tolerability of tapentadol immediate release in patients with lower back pain or osteoarthritis of the hip or knee over 90 days: a randomized, double-blind study. Curr Med Res Opin. 2009 May;25(5):1095-104. doi: 10.1185/03007990902816970. PMID: 19301989.
9. Imanaka K et al. Efficacy and safety of oral tapentadol extended release in Japanese and Korean patients with moderate to severe, chronic malignant tumor-related pain. Curr Med Res Opin. 2013 Oct;29(10):1399-409. doi: 10.1185/03007995.2013.831816. Epub 2013 Aug 23. PMID: 23937387.
10. Niesters M et al. Tapentadol potentiates descending pain inhibition in chronic pain patients with diabetic polyneuropathy. Br J Anaesth. 2014 Jul;113(1):148-56. doi: 10.1093/bja/aeu056. Epub 2014 Apr 8. PMID: 24713310.
11. Serrie A, Lange B, Steup A. Tapentadol prolonged-release for moderate-to-severe chronic osteoarthritis knee pain: a double-blind, randomized, placebo- and oxycodone controlled release-controlled study. Curr Med Res Opin. 2017 Aug;33(8):1423-1432. doi: 10.1080/03007995.2017.1335189. Epub 2017 Jun 11. PMID: 28537501.
12. Steigerwald I et al. Effectiveness and tolerability of tapentadol prolonged release compared with prior opioid therapy for the management of severe, chronic osteoarthritis pain. Clin Drug Investig. 2013 Sep;33(9):607-19. doi: 10.1007/s40261-013-0102-0. PMID: 23912473; PMCID: PMC3751342.
13. Tominaga Y, et al. Methodological Issues in Conducting Pilot Trials in Chronic Pain as Randomized, Double-blind, Placebo-controlled Studies. Drug Res (Stuttg). 2016 Jul;66(7):363-70. doi: 10.1055/s-0042-107669. Epub 2016 May 25. PMID: 27224908.
14. Vinik AI et al. A randomized withdrawal, placebo-controlled study evaluating the efficacy and tolerability of tapentadol extended release in patients with chronic painful diabetic peripheral neuropathy. Diabetes Care. 2014 Aug;37(8):2302-9. doi: 10.2337/dc13-2291. Epub 2014 May 21. PMID: 24848284.
15. Vorsanger G et al. Post hoc analyses of data from a 90-day clinical trial evaluating the tolerability and efficacy of tapentadol immediate release and oxycodone immediate release for the relief of moderate to severe pain in elderly and nonelderly patients. Pain Res Manag. 2011 Jul-Aug;16(4):245-51. doi: 10.1155/2011/323985. PMID: 22059194; PMCID: PMC3202376.
16. Wild JE et al. Long-term safety and tolerability of tapentadol extended release for the management of chronic low back pain or osteoarthritis pain. Pain Pract. 2010 Sep-Oct;10(5):416-27. doi: 10.1111/j.1533-2500.2010.00397.x. PMID: 20602712.


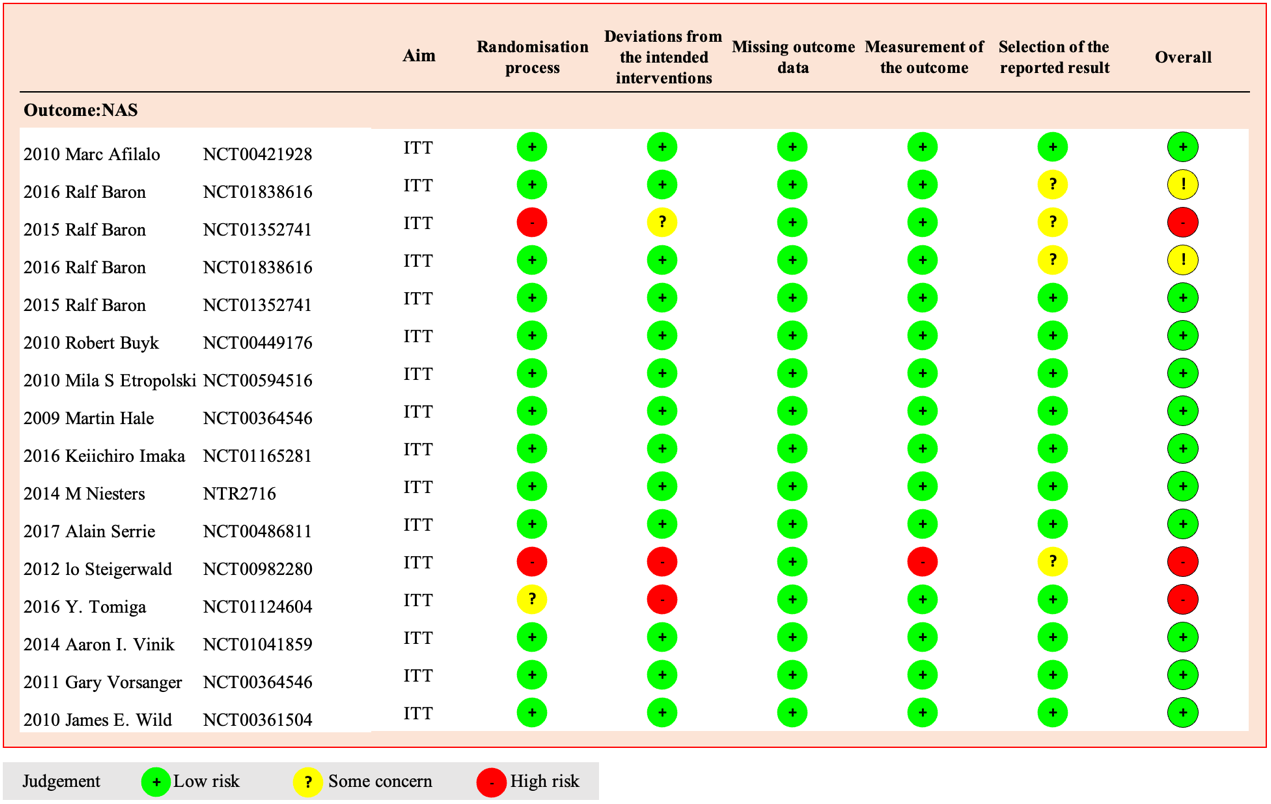


## Figure S1. Risk of Bias

In this color-coded ranking, green color represents low risk of bias, yellow some concerns, and red high risk of bias.


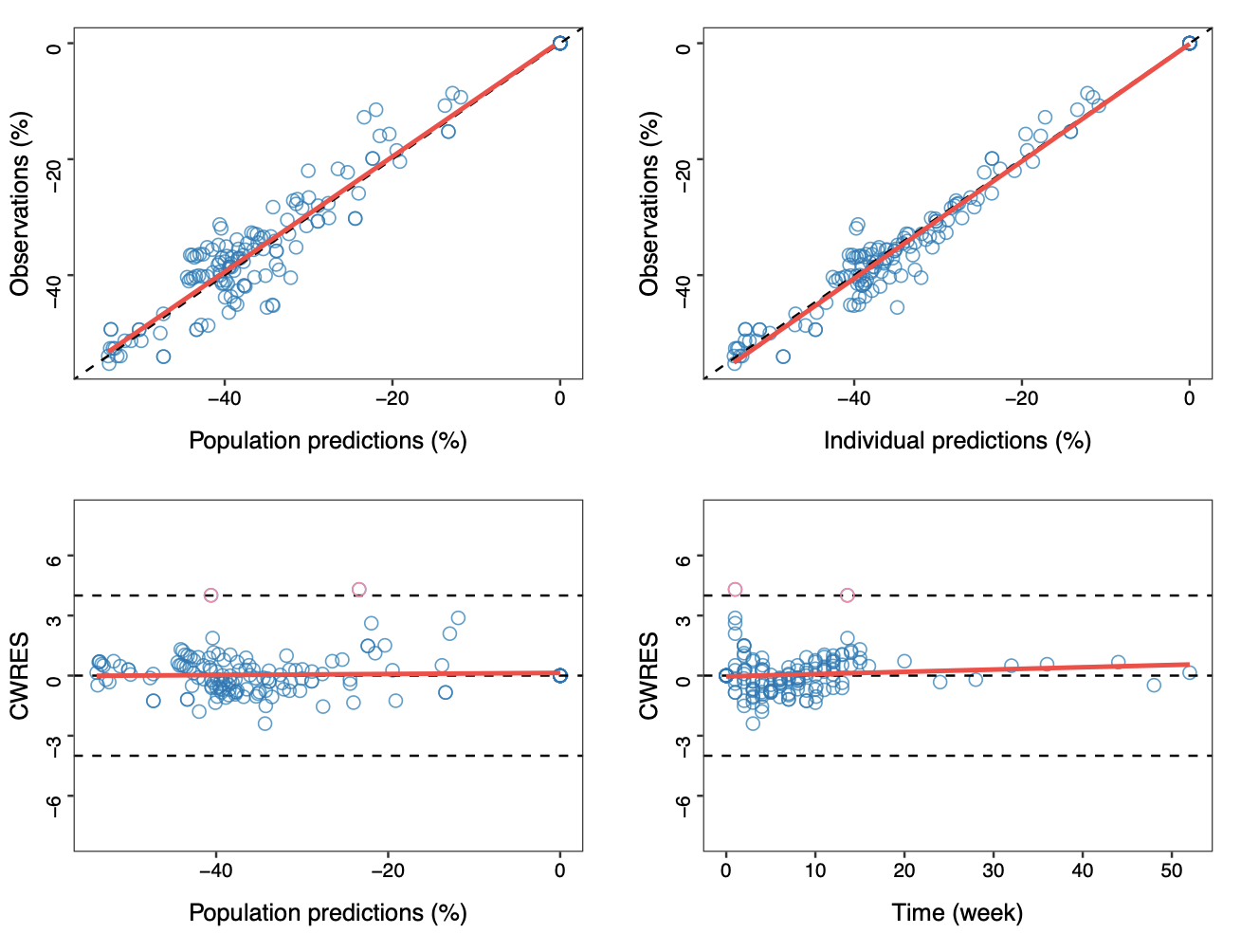


## Figure S2. Goodness of fit

Points represent individual data points; red lines represent local regression smoothing lines; solid black line is the line of identity; dashed black lines represent 95% CI. CWRES conditional weighted residuals, CI confidence interval.

## Table S2. Typical time (week) to achieve -30% efficacy

| **Age (year)** | **Published on or before 2014** | | **Published after 2014** | |
| --- | --- | --- | --- | --- |
|  | **placebo controlled** | **non-placebo controlled** | **placebo controlled** | **non-placebo controlled** |
| 45 | 5.6 | 2.5 | 10.0 | 6.9 |
| 46 | 5.1 | 2.3 | 9.1 | 6.3 |
| 47 | 4.7 | 2.1 | 8.4 | 5.8 |
| 48 | 4.3 | 2.0 | 7.7 | 5.4 |
| 49 | 4.0 | 1.8 | 7.2 | 5.0 |
| 50 | 3.8 | 1.7 | 6.7 | 4.7 |
| 51 | 3.5 | 1.6 | 6.3 | 4.4 |
| 52 | 3.3 | 1.5 | 5.9 | 4.1 |
| 53 | 3.1 | 1.4 | 5.6 | 3.9 |
| 54 | 3.0 | 1.3 | 5.3 | 3.7 |
| 55 | 2.8 | 1.3 | 5.0 | 3.5 |
| 56 | 2.7 | 1.2 | 4.8 | 3.3 |
| 57 | 2.6 | 1.2 | 4.6 | 3.2 |
| 58 | 2.4 | 1.1 | 4.4 | 3.0 |
| 59 | 2.3 | 1.1 | 4.2 | 2.9 |
| 60 | 2.2 | 1.0 | 4.0 | 2.8 |
| 61 | 2.2 | 1.0 | 3.9 | 2.7 |
| 62 | 2.1 | 0.9 | 3.7 | 2.6 |
| 63 | 2.0 | 0.9 | 3.6 | 2.5 |
| 64 | 1.9 | 0.9 | 3.4 | 2.4 |
| 65 | 1.9 | 0.8 | 3.3 | 2.3 |

## **Reference**

1. Meibohm B, Derendorf H. Basic concepts of pharmacokinetic/pharmacodynamic (PK/PD) modelling. Int J Clin Pharmacol Ther. 1997 Oct;35(10):401-13. PMID: 9352388.
2. Irby DJ, Ibrahim ME, Dauki AM, Badawi MA, Illamola SM, Chen M, Wang Y, Liu X, Phelps MA, Mould DR. Approaches to handling missing or "problematic" pharmacology data: Pharmacokinetics. CPT Pharmacometrics Syst Pharmacol. 2021 Apr;10(4):291-308. doi: 10.1002/psp4.12611. PMID: 33715307; PMCID: PMC8099444.
3. Zhu H, Liu H, Sui Z, Yu J, Zheng Q, Li L. Quantitative comparison of different inhaled corticosteroids in the treatment of asthma in children. Pediatr Res. 2023 Jan;93(1):31-38. doi: 10.1038/s41390-022-02095-8. Epub 2022 May 11. PMID: 35545660.
4. Ette EI, Ludden TM. Population pharmacokinetic modeling: the importance of informative graphics. Pharm Res. 1995 Dec;12(12):1845-55. doi: 10.1023/a:1016215116835. PMID: 8786955.
5. Holford NH, Chan PL, Nutt JG, Kieburtz K, Shoulson I; Parkinson Study Group. Disease progression and pharmacodynamics in Parkinson disease - evidence for functional protection with levodopa and other treatments. J Pharmacokinet Pharmacodyn. 2006 Jun;33(3):281-311. doi: 10.1007/s10928-006-9012-6. Epub 2006 Apr 20. PMID: 16625427.
6. Kuti JL, Dandekar PK, Nightingale CH, Nicolau DP. Use of Monte Carlo simulation to design an optimized pharmacodynamic dosing strategy for meropenem. J Clin Pharmacol. 2003 Oct;43(10):1116-23. doi: 10.1177/0091270003257225. Erratum in: J Clin Pharmacol. 2005 Mar;45(3):357. PMID: 14517194.
7. Ette EI, Williams PJ, Kim YH, Lane JR, Liu MJ, Capparelli EV. Model appropriateness and population pharmacokinetic modeling. J Clin Pharmacol. 2003 Jun;43(6):610-23. PMID: 12817524.
